# Mechanism of tonifying-kidney Chinese herbal medicine in the treatment of chronic heart failure

**DOI:** 10.3389/fcvm.2022.988360

**Published:** 2022-09-12

**Authors:** Lizhen Chen, Dayun Yu, Shuang Ling, Jin-Wen Xu

**Affiliations:** Institute of Interdisciplinary Medical Science, Shanghai University of Traditional Chinese Medicine, Shanghai, China

**Keywords:** chronic heart failure, classical prescription, inflammation, neurohumoral factors, energy metabolism

## Abstract

According to traditional Chinese medicine (TCM), chronic heart failure has the basic pathological characteristics of “heart-kidney yang deficiency.” Chronic heart failure with heart- and kidney-Yang deficiency has good overlap with New York Heart Association (NYHA) classes III and IV. Traditional Chinese medicine classical prescriptions for the treatment of chronic heart failure often take “warming and tonifying kidney-Yang” as the core, supplemented by herbal compositions with functions of “promoting blood circulation and dispersing blood stasis.” Nowadays, there are still many classical and folk prescriptions for chronic heart failure treatment, such as Zhenwu decoction, Bushen Huoxue decoction, Shenfu decoction, Sini decoction, as well as Qili Qiangxin capsule. This review focuses on classical formulations and their active constituents that play a key role in preventing chronic heart failure by suppressing inflammation and modulating immune and neurohumoral factors. In addition, given that mitochondrial metabolic reprogramming has intimate relation with inflammation, cardiac hypertrophy, and fibrosis, the regulatory role of classical prescriptions and their active components in metabolic reprogramming, including glycolysis and lipid β-oxidation, is also presented. Although the exact mechanism is unknown, the classical TCM prescriptions still have good clinical effects in treating chronic heart failure. This review will provide a modern pharmacological explanation for its mechanism and offer evidence for clinical medication by combining TCM syndrome differentiation with chronic heart failure clinical stages.

## Introduction

The word “heart failure” has been recorded in the ancient Chinese medicine book “Huangdi Neijing” more than 2,300 years ago. According to the combination of different traditional Chinese medicine (TCM) syndrome elements, TCM can divide chronic heart failure into 4–6 TCM syndromes (1), which are mainly related to deficiency of heart and kidney yang and heart qi deficiency. The report of Shi et al. (2) compared the main TCM syndromes of chronic heart failure with the New York Heart Association (NYHA) classification, and found that there is a certain correlation between NYHA classification and the distribution of heart-kidney yang deficiency and heart-qi deficiency. Heart-qi deficiency is basically concentrated in categories I and II, and the frequency of kidney yang deficiency and body fluid retention in class III+IV is higher than that in class I and II. Obviously, in the NYHA classification, heart-kidney-yang deficiency is the stage where heart failure progresses into a more severe stage (2). Common classic and folk prescriptions for the treatment of chronic heart failure include Zhenwu decoction, Lingguizhugan decoction, Sini decoction, Shenfu decoction, Bushen Huoxue decoction, and Qili Qiangxin capsule, etc. (Table 1). Their effectiveness on chronic heart failure has been proved by increasing clinical investigations (3–5). They are superior to Western medicine alone in terms of treatment efficacy, whether judged by TCM symptom efficacy or by NYHA functional classification (Supplementary Table 1). The RCRI/Lee's score, 6MWD, LVEF, and CCE were also better to western medicine alone on the endpoints of chronic heart failure (Supplementary Table 1). In terms of blood parameters related to cardiac function and inflammation, such as NT-proBNP, cTnI, sST2, CK-MB, and hs-CRP, the combination of classical prescriptions also showed more favorable improvement in chronic heart failure (Supplementary Table 1).

**Table 1:** Composition of several classical prescriptions for treatment of chronic heart failure.

**Name of classical prescription**	**Source**	**Years**	**Major composition and efficacy**			
			**Warming and tonifying kidney-Yang**	**Promoting blood circulation and dispersing blood stasis**	**Dredge meridians and Qi activity**	**Relieving diuresis and eliminating dampness**
Jinkui Shenqi pill	Zhongjing Zhang, “Synopsis of the Golden Chamber”	200–210 A.D.	Aconite, Cassia Twig	Chinese Peony Bark	Rehmannia root, Chinese Yams	Rhizoma Alismatis, Tuckahoe
Zhenwu decoction	Zhongjing Zhang, “Treatise on Febrile Diseases”	205 A.D.	Aconite, Ginger	Radix Paeoniae Rubra	Atractylodes Rhizome	Tuckahoe
Lingguizhugan decoction	Zhongjing Zhang, “Synopsis of the Golden Chamber”	200–210 A.D.	Cassia Twig	N/A	Atractylodes Rhizome, Baked licorice	Tuckahoe
Sini powder	Zhongjing Zhang, “Treatise on Febrile Diseases”	205 A.D.	Aconite, Dried Ginger	Licorice	N/A	N/A
Shenfu decoction	Yonghe Yan, “Renewed Yan's Jisheng recipes”	1253 A.D	Aconite	N/A	Ginseng	N/A
BuShen HuoXue decoction	Lian Zhao, “Great Achievement of Traumatology”	1891 A.D.	Fructus Corni, Chinese Dodder Seed, Herba Cistanches, Fructus Psoraleae, Cortex Eucommiae	Safflower, Chinese Angelica, Myrrha	Prepared rehmannia root, Chinese wolfberry	Radix angelicae tuhuo
Qili Qiangxin capsule	Chinese Pharmacopeia 2015 edition, Volume I	2015 A.D.	Aconite, Cassia Twig	Safflower, Danshen Root	Astragalus Root, Ginseng, radix *Polygonati officinalis*	Flixweed Tansy Mustard Seeds, Rhizoma Alismatis, Chinese Silkvine Root-bark, Dried tangerine

The occurrence and development of chronic heart failure includes a variety of processes, which are related to inflammation of heart tissue, participation of immune cells (macrophages, lymphocytes, and neutrophils, etc.), regulation of neurohumoral factors, and programmed cell death (apoptosis, pyroptosis, etc.), myocardial hypertrophy, and cardiac fibrosis (6–8). Experimental investigations in animals have confirmed that a lot of classic prescriptions for tonifying kidney can improve inflammation (9, 10), oxidative stress and cellular injury (11), inhibit cardiomyocyte death (10, 11), and myocardial hypertrophy (12–14), regulate neurohumoral factors (15, 16), and alleviate cardiac fibrosis (17–20). Accumulating studies have been reported that (1) Lingguizhugan decoction and Zhenwu decoction prevent cardiac hypertrophy by inhibiting MAP kinase activity (12, 13); (2) Lingguizhugan decoction improves ventricular hypertrophy by restraining NLRP3/Caspase-1/GSDMD inflammation and pyroptosis pathways (10); (3) Lingguizhugan decoction also antagonizes oxidative stress injury through the Nrf2/Keap1/HO-1 pathway (11); (4) Shenfu recipe mitigates heart failure by activating AMPK-mediated metabolism of fatty acids and glucose in the heart (17); (5) Shenfu injection enhances eNOS activity through PI3K/Akt signaling pathway, promotes vasodilation and improves microcirculation of failing heart (21); (6) Sini Decoction significantly decreases the levels of hs-CRP, TNF-α, IL-6, and IL-1β in rat myocardial tissue, reduces the levels of renin, angiotensin II, aldosterone, ANP, and ET-1 in plasma, and limits the vascular inflammation of the heart (9, 15); (7) Sini decoction also reduces TLR-2/4 and TGF-β1 levels to improve early ventricular remodeling and cardiac function after myocardial infarction (18); (8) Qili Qiangxin capsule alleviates cardiac remodeling by inhibiting TGF-β1/Smad3 and NF-κB signaling pathways (19).

The levels of circulating pro-inflammatory biomarkers are closely related to disease severity and prognosis in patients with heart failure. Despite disappointing clinical trial results inflammation is still considered to be a major pathophysiological factor contributing to chronic heart failure, including heart failure with reduced ejection fraction and heart failure with preserved ejection fraction (6, 22). Moreover, inflammation and metabolic reprogramming are mutually regulated objects. For example, LPS can enhance the activity of HIF-1 and participate in the induction of glycolysis and pro-inflammatory genes, especially IL-1β. In contrast, the tricarboxylic acid cycle (TAC) intermediate, succinic acid, and itaconic acid, a derivative of citric acid, have pro- and anti-inflammatory effects, respectively (23–25). Therefore, metabolic reprogramming during heart failure has become a hotspot of research in recent years. Given that mitochondria are key subcellular organelles of cardiomyocytes, important discoveries have been made in mitochondrial energy, glucose, fatty acid, and amino acid metabolic reprogramming (also known as metabolic remodeling) in heart failure, which will be targets for next-generation treatments (26, 27). Heart failure is also a bioenergetic disease. In addition to cardiac hypertrophy (28, 29), metabolic reprogramming also occurs during cardiac fibrosis (30, 31). Last but not the least, neurohumoral factors, such as the renin-angiotensin-aldosterone system, are inducers of chronic heart failure and low-grade inflammation. Indeed, antagonism of neurohormonal systems is a key therapy for heart failure. In brief, this review focuses on the modern medical characteristics of classic kidney-tonifying prescriptions for the treatment of chronic heart failure, and discusses the classic and modern pharmacological mechanisms of active ingredients on inflammation, neurohumoral factors, and metabolic reprogramming.

## Immunity and inflammation in the heart- and kidney-Yang deficiency

Insufficient tissue oxygen and nutrient supply, accumulated harmful metabolites and cell death are the main mechanisms of heart failure with reduced ejection fraction (HFrEF). HFrEF happens because of chronic low-grade inflammation and immune activation, tissue fibrosis, and remodeling, decreased ventricular compliance caused by chronic myocardial machinery overwork. The importance of inflammation in heart failure has been summarized, analyzed, and prospected by many excellent reviews (32–34), and we will not repeat them here. Now many kinds of immune cells, such as NK cells, T cells, B cells, monocytes, macrophages, are known to participate in the process of heart failure. CD4^+^ T lymphocytes are progressively augmented and broadly activated in circulation, failing heart and spleen, and are also necessary for pathological left ventricular remodeling in chronic heart failure (35). Treg, CD4^+^, CD8^+^ T cells, and the ratio of CD4^+^ to CD8^+^ T cells are expected to be biomarkers of heart failure (36). The plasticity of monocytes and the heterogeneity of monocyte-derived macrophages have important implications for cardiac remodeling. After myocardial injury, the medulla and extramedullary hematopoiesis enlarges, the recruitment of cardiac monocytes CCR2^+^ increases, and the enhanced cell wall tension stimulates the proliferation of local macrophages in the heart (37). Otherwise, patients with advanced heart failure have a high IL-6 level produced by peripheral blood monocytes, a low level of circulating NK cell number, and impaired cytolytic function of NK cells on IL-2 and IL-12. Furthermore, the impaired lytic function is closely related to the level of IL-6 produced by unstimulated peripheral blood mononuclear cells (38).

**Figure 1:**
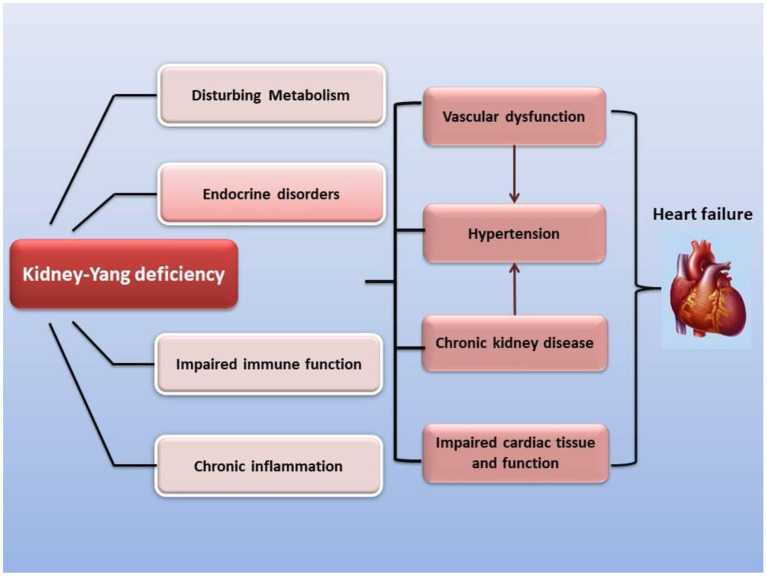
The modern medical understanding that kidney-Yang deficiency promotes the development of chronic heart failure. Kidney-Yang deficiency leads to metabolic disturbances, endocrine disorders, immune function imbalance, and low-grade chronic inflammation, which further contributes to vascular dysfunction, hypertension, chronic kidney disease, and cardiac tissue and functional imbalance, and eventually guides to chronic heart failure.

According to previous reports, inflammation and immune disorders are important factors for heart failure with kidney-yang deficiency. Many Chinese herbal compounds can alleviate these inflammation and immune system disorders. The following are some research reports. After Shenfu Injection was used to treat 56 patients with heart failure and Kidney-Yang deficiency, the increased levels of CD4^+^CD25^+^Foxp3^+^Treg and CD4^+^ T cells were suppressed, while the low level of IL-10 was elevated (39). In an early clinical study of 57 elderly people with kidney deficiency, the percentage of OKT4/CD4^+^ in the elderly was significantly lower than that of the control group, whereas OKT8/CD8^+^ was remarkably increased, and as a result the ratio of OKT4/OKT8 was also markedly decreased compared with the control group (40). Furthermore, their immunological changes have also been confirmed in rat and mouse models of kidney-Yang deficiency (Supplementary Table 2). Many cellular immune parameters are suppressed, such as thymus index, spleen index, T lymph and B lymph stimulation index, NK cell activity, macrophage phagocytosis, etc. (Supplementary Table 2). As the number of CD4^+^ cells decreases and the number of CD8^+^ cells increases, the ratio of CD4^+^/CD8^+^ is also reduced. The serum levels of IL-4 and the ratio of IL-4/IFN-γ are greatly elevated (Supplementary Table 2), yet the trend of the serum IL-2 and IFN-γ levels are contrary to the above. A variety of prescriptions for improving kidney-Yang deficiency, such as anti-aging tablet, Jinkui Shenqi pill and Yougui pill, reversed these cellular and humoral immune indicators (Supplementary Table 2). Kidney-Yang deficiency aggravates the inflammatory response of the heart, including the activated NF-κB pathway of myocardium, the elevated activities of ERK1/2, p38, and JNK in the tissues, the upregulated JAK2/STAT3 signal, and the unbalanced homeostasis of endoplasmic reticulum stress (19, 41–44). The application of prescriptions for resisting kidney-Yang deficiency can reverse the failure of heart function (Figure 1).

In the past 3 years, many studies have explored the mechanism of Zhenwu decoction on chronic heart failure by using network pharmacology methods, which revealed many signaling pathways, key target genes and key active ingredients of Zhenwu decoction. These studies have found that Zhenwu Decoction has multiple key signaling pathways for improving heart failure, including inflammation-related MAPK, NF-κB, IL-17, TNF-α, HIF-1α, TLR, and PPARG signaling pathways, as well as platelet activation and arachidonic acid metabolism. It also involves apoptosis, p53 signaling, adrenergic signaling in cardiomyocytes, renin-angiotensin system, and Insulin resistance related to myocardial damage, function, and fibrosis, in addition to calcium signaling and cGMP-PKG signaling related to cardiac contraction and relaxation (Supplementary Table 3). In the search for key target genes, the authors found that inflammation-related IL-6, TNF-α, PTGS2, PPARG, IKBKB, oxidative stress-related GSTP1, GSTM1, GSTM2, cell death-related CASP3, CASP8, CASP9, BCL2, and various receptors that affected myocardial function, such as adrenoceptor alpha subtype (ADRA2A, ADRA2B, and ADRA1A), androgen receptor (AR), estrogen receptor (ESR1), angiotensin II receptor 1 (AGTR1), epidermal growth factor receptor (EGFR), and cholinergic receptor muscarinic 1 (CHRM1), were targeted by Zhenwu decoction (Supplementary Table 3). Moreover, β-sitosterol, stigmasterol, kaempferol, norcoclaurine, 3β-acetoxyatractylone, paeoniflorin, (+)-catechin, hederagenin, and trametenolic acid were identified as key active ingredients of Zhenwu decoction (Supplementary Table 3). These findings indicated that the main active components in Zhenwu decoction can effectively act on key genes related to heart failure through molecular docking detection (Supplementary Table 3).

## Chinese herbal medicinal ingredients for warming and tonifying kidney-Yang target involved in anti-inflammatory and cardiac function homeostasis

Traditional Chinese medicine herbs for warming and tonifying kidney-Yang account for a majority of all traditional and folk prescriptions for chronic heart failure treatment. Therefore, this section will discuss the effects of their active components on anti-inflammatory and immunomodulation. The main active ingredients are listed in Supplementary Table 4.

Higenamine, an active component of *Aconitum carmichaeli*, has shown a positive effect on improving chronic heart failure. Higenamine enhanced contractility of failing hearts and improved energy metabolism in cardiomyocytes, inhibited TGF-β1/Smad signaling transduction, thereby reducing cardiac fibrosis caused by cardiac fibroblast proliferation (45, 46). The activation of the NF-κB increases the production of inflammatory cytokines and chemokines. In previous studies, higenamine inhibited the activation of NF-κB, the expression of COX-2, iNOS, TNF-α, and IL-6, and the production of PGE2 and NO through LPS and IL-1β stimulation (47–49). Similarly, monkshood polysaccharide also had the effect of inhibiting inflammation by blocking NF-κB pathway, and prevented the expression of iNOS and inflammatory cytokines (TNF-α, IL-1β, and IL-6) (50). Macrophages and lymphocytes are the most common immune cells. In a murine model of spinal cord injury, Higenamine was found to promote the activation of M2 macrophages and reduce the production of HMGB1 through HO-1 induction (51). Other studies have also shown that monkshood polysaccharides inhibited the proliferation of splenic lymphocytes induced by LPS or ConA *in vivo* and *in vitro*, and the production of antibody *in vivo* in mice (52). Cinnamaldehyde, an important active ingredient of *Cinnamomum cassia* Presl., is also often used as a condiment in beverages, cakes, and perfumes. Previous studies have supported that Cinnamaldehyde attenuated cardiac hypertrophy and fibrosis caused by pressure overload (53) and acute myocardial ischemia induced by isoproterenol (54). Cardiac hypertrophy is a systemic chronic inflammation, while myocardial ischemia initiates an intense inflammatory response. The possible mechanisms by which cinnamaldehyde protects the heart are anti-inflammation. Numerous studies have demonstrated that cinnamaldehyde regulates the NF-κB, p38, JNK, and Jak/Stat pathways to inhibit expressions of IL-6, IL-8, and TNF-α induced by IL-1β (55) and MCP-1, MMP-2, and LOX-1 induced by ox-LDL (56). Cinnamaldehyde also ameliorated LPS-induced cardiac dysfunction by inhibiting ROS production and autophagy through TLR4-NOX4 pathway (57), suppressed fructose-induced cardiac inflammation and fibrosis by attenuating CD36-mediated TLR4/6 and IL-1R-associated kinase 4/1 signaling (58). In addition, the accumulation of succinate in cytoplasm stimulated HIF-1α activity, which activated the expression of NLRP3. Cinnamaldehyde significantly reduced synovial inflammation in rheumatoid arthritis rats by blocking the maturation of IL-1β derived from NLRP3 (59). Cornuside and morroniside are major active components of *Cornus officinalis*. Cornuside and morroniside have been reported to reduce heart damage caused by ligation of anterior descending coronary artery or ischemia-reperfusion (60, 61). The underlying mechanisms are that cornuside and morroniside significantly inhibit the expression of iNOS and COX-2 induced by LPS, and production of TNF-α, IL-1β, IL-6, NO, and PGE2 through the regulation of TLR4/NF-κB, ERK1/2, p38, JNK, STAT3, and Nrf2/HO-1 signaling pathways (62–64).

Psoraleae Fructus is a Traditional Chinese herb with the effect of warming kidney and enhancing yang. It contains terpenoids, coumarins, isopentenyl flavonoids, chalcones, and other active components, such as bakuchiol, psoralen, isopsoralen, corylifol A (also known as corylinin), neobavisoflavone, and isobavachalcone. It has been reported that bakuchiol in studies can attenuate myocardial ischemia-reperfusion injury, pathological myocardial hypertrophy, and heart failure (65, 66). Bakuchiol, psoralen, and isopsoralen demonstrated the effect of stimulating vasodilation through endothelium-dependent and endothelium-independent pathways (67). Other active constituents in Psoraleae Fructus such as Psoralen, corylifol A, neobavisoflavone, and bakuchiol inhibited the expression and secretion of TNF-α, IL-1β, and IL-6 (68). Bakuchiol also blocked NF-κB pathway activity (68).

Epimedium, Eucommia, Cistanche, and Cnidium are all important herbs for warming and tonifying kidney-Yang. Many studies have revealed that their active ingredients, such as icariin, icariside II, aucubin, echinacoside, and osthole improved myocardial hypertrophy, remodeling and fibrosis induced by pressure overload (69–73), or myocardial injury and remodeling caused by isoproterenol (74, 75). Moreover, icariin, syringaresinol, and osthole also prevented myocardial ischemia/reperfusion injury (76–78), and aucubin significantly reduced myocardial infarction-induced cardiac remodeling (79). Overall, accumulating reports indicate that icariin, icariside II, aucubin, echinacoside, and osthole all inhibited the activity of NF-κB (71, 73, 80–84). Other reports also pointed out that icariin blocked JNK (80), echinacoside prevented STAT3 (83), and echinacoside and aucubin reduced activity of inflammasome NLRP3 (75, 85). To sum up, the active ingredients of herbs for warming and tonifying Kidney-Yang basically demonstrated anti-inflammatory ability.

## Effect of prescriptions on changes of sympathetic excitability and neurohumoral factors in chronic heart failure

**Figure 2:**
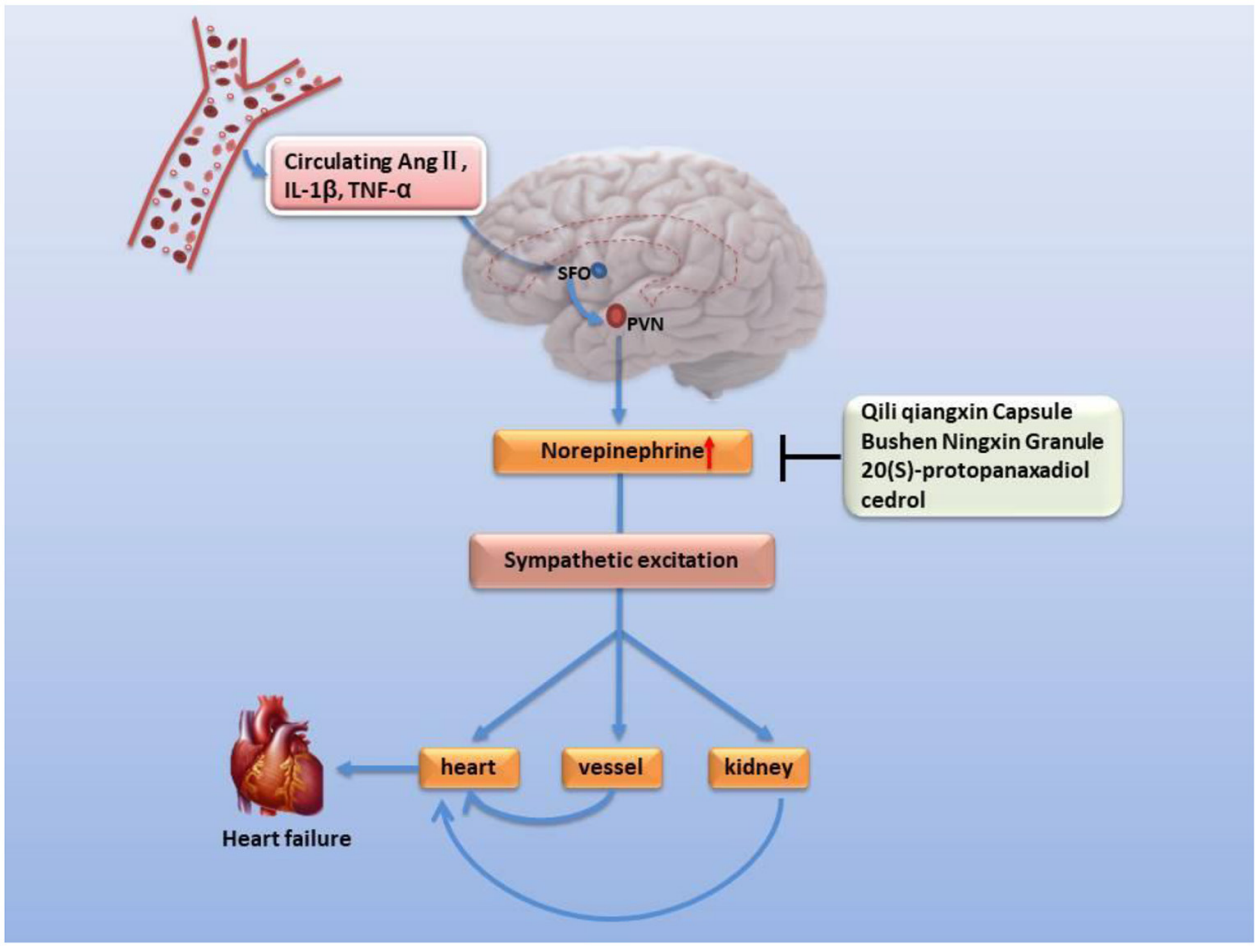
Inhibitory effect of classic formulations and their active ingredients on norepinephrine secretion and neural excitability in the hypothalamus. Circulating pro-inflammatory factors enhance sympathetic excitability and the risk of heart failure. SFO's perception of circulating proinflammatory factors leads to increased secretion of norepinephrine in PVN neurotransmitters and promotes sympathetic excitability, resulting in microvascular contraction, renal fluid retention and increased renin release, raising the level of angiotensin II and aldosterone in blood, elevating heart rate and arrhythmia, and causing left ventricular hypertrophy.

Sympathetic hyperactivity is an identified hallmark of chronic heart failure. Cardiac sympathetic afferent reflex (CSAR), a sympathetic excitatory reflex with positive feedback characteristics, can be activated by endogenous substances during chronic heart failure, such as Ang II, AT1 receptor, NF-κB, and NADPH oxidase pathways (86–88). The pathological enhancement of CSAR is one of the major reasons for sympathetic excitation in chronic heart failure. The paraventricular nucleus (PVN) of the hypothalamus is an important component of the central pathway of the CSAR, and also plays a key center for integration of neuroendocrine and sympathetic nerve activities. The subfornical organ (SFO), as a periventricular structure, senses circulating IL-1β, TNF-α, and Ang II, elevates sympathetic excitability and transmits it to the downstream PVN (89–91). In addition, Ang II has been reported to promote the release and synthesis of PVN's excitatory neurotransmitter norepinephrine (92), enhancing the plasma norepinephrine level and eventually leading to impaired cardiac function in heart failure (90, 93). Furthermore, compared with normal rats, the norepinephrine gene expression and plasma concentration in the hypothalamus of rats with Yang deficiency reached a high level, suggesting its sympathetic hyperactivity (94). Two tonifying kidney-Yang prescriptions, Qili Qiangxin Capsule and Bushen Ningxin Granule, can downregulate the expression and secretion of norepinephrine in the hypothalamus due to heart failure or anxiety (95, 96) (Figure 2). For example, the 20(S)-protopanaxadiol of Ginseng in Qili Qiangxin Capsule, and the cedrol of Boziren in Bushen Ningxin Granules, reduce levels of norepinephrine in the hippocampus of rodents (97, 98) (Supplementary Table 5).

**Figure 3:**
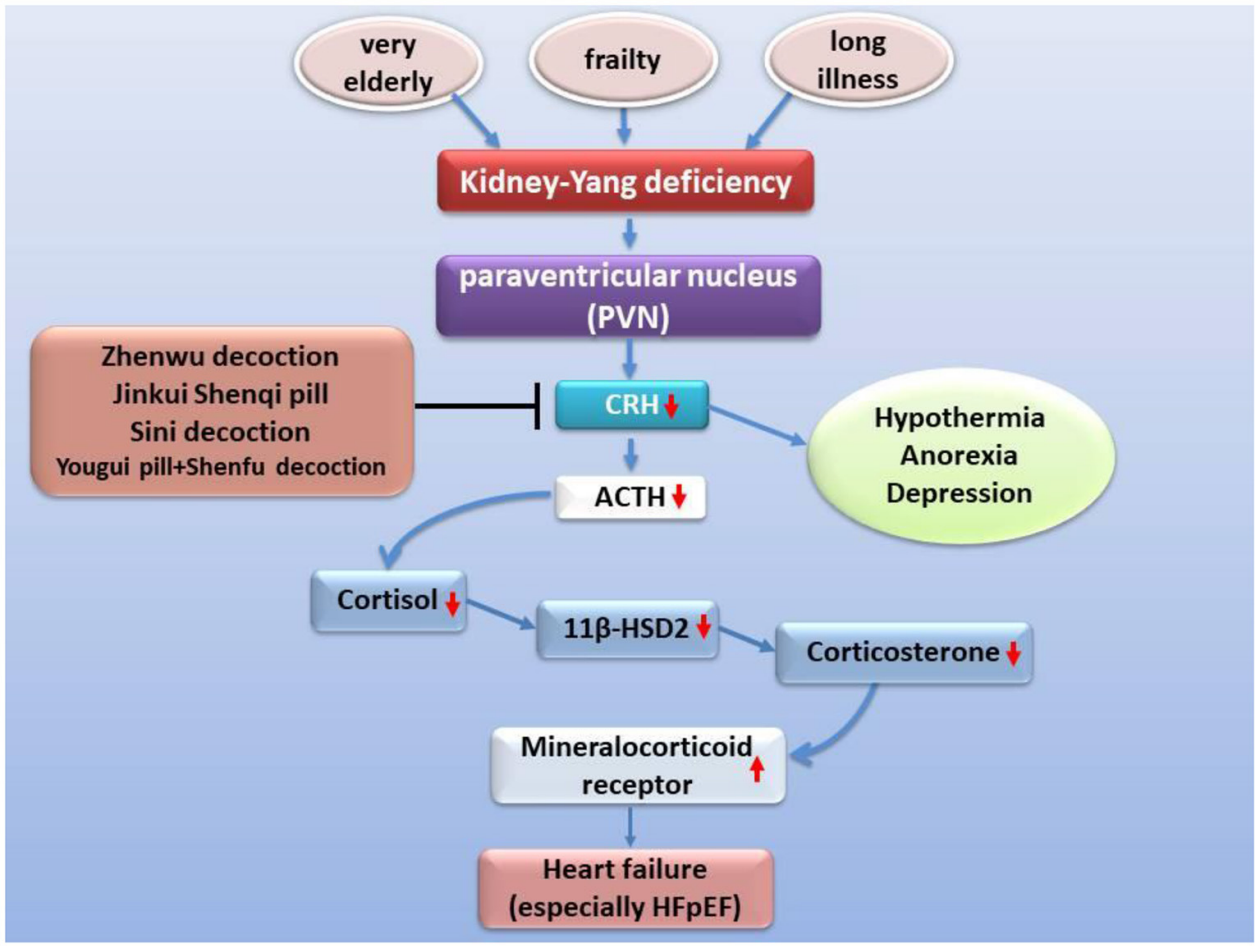
Improvement of heart failure by classic prescription via HPA axis. Kidney Yang deficiency is linked to heart failure via the hypothalamic-pituitary-adrenal axis. Under the condition of kidney-Yang deficiency driven by factors such as advanced age, the secretion of CRH secreted by PVN decreases, the activation of the hypothalamic-pituitary-adrenal axis is inhibited, the expression of 11β-HSD2 drops while the expression of mineralocorticoid receptor (MR) rises, cardiac hypertrophy and fibrosis, and functional events increased.

Corticotropin-releasing hormone (CRH) is one of a variety of neuroendocrine hormones secreted by PVN. The expression of CRH in PVN of rats with ischemia-induced heart failure is elevated with the increased expression of pro-inflammatory cytokines and pro-inflammatory genes, even up to two times (99, 100). Corticotropin-releasing hormone has been reported to enhance the excitability of hypothalamic sympathetic nerves in heart failure (101). However, the function of the hypothalamic-pituitary-adrenal axis decreases with age, especially in very elderly patients with heart failure, the gene expression and secretion of related neurohumoral declines, such as CRH (102, 103), 11β-HSD2 (104). Due to negative feedback, the expression and activity of mineralocorticoid receptor (MR) increased instead (105). Moreover, it is worth noting that chronic heart failure patients based on NYHA classes III and IV have a higher proportion of hypothermia (106, 107), anorexia (108, 109), depression (110, 111), and a poor prognosis, which is similar to the phenomenon of kidney-Yang deficiency of TCM (112) or a lower response of ACTH and cortisol (102, 113). Many classic prescriptions for warming and nourishing kidney-Yang, such as Sini decoction reverses hypothermia and balances hypothalamic-pituitary-adrenal axis *in vivo* (114–117) and improve chronic heart failure (38, 114, 118) (Figure 3). Urotensin II is a homolog of corticotropin-releasing factor, and its tissue UTR2 distribution is highly consistent with its receptor (119, 120). The activity of urotensin is mainly regulated by autocrine and paracrine mechanisms. Urotensin is a strong vasoconstrictor and proinflammatory mediator in patients with heart failure, which can lead to cardiac fibrosis, myocardial hypertrophy, and remodeling (121–124). These characteristics can explain the clinical findings related to heart failure. The plasma urotensin level of patients with heart failure is much higher than healthy individuals (125, 126). Studies have revealed that the traditional prescription Shenfu injection and Fuling Sini decoction effectively reduce the plasma urotensin levels in patients with chronic heart failure (127, 128). In addition, vasopressin (AVP) is another neurohumoral factor, which is secreted by magnocellular neurosecretory neurons in the hypothalamic supraoptic nucleus and PVN, and regulates blood pressure, hyponatremia, and edema associated with heart failure (129, 130). According to the dialectical classification of TCM, the expression levels of AVP and AQP-2 are much higher in patients with Yang deficiency and water retention syndrome (equivalent to NYHA class III+IV) than in those with both Qi and Yin deficiency syndrome (equivalent to NYHA class II) (131). The warming Yang prescriptions, such as Qili Qiangxin capsule, Shenfu injection, and Zhenwu decoction, can reduce plasma AVP levels, AVP V2 receptor and AQP-2 expression in renal tissue, and improve hemodynamics and cardiac function (16, 132, 133).

The renin-AngII-aldosterone system is an endocrine axis that maintains blood pressure and body fluid homeostasis. Homeostatic imbalance is directly involved in the various processes required for the progression of heart failure: oxidative stress, inflammation, cardiac hypertrophy, cell death, and tissue fibrosis. Multiple clinical investigations of hospitalized patients have found that elderly patients (average age about 73 years) with heart failure and kidney-Yang deficiency have higher plasma AngII level, slightly lower left ventricular ejection fraction (EF) and fractional shortening (FS), and much lower left ventricular diastolic function (E/A ratio) than heart failure patients with non-kidney-Yang deficiency (2, 5, 134). Moreover, patients with kidney-Yang deficiency are mostly distributed in NYHA class III and IV, and non-kidney-Yang deficiency exists in NYHA class II (2, 5, 134). It has been proved in animal experiments that a variety of herbal formulas, including Sini decoction, Qili Qiangxin capsules, and Shenfu Qiangxin Mixture, can effectively reduce the plasma Ang II and aldosterone level in rats in a dose-dependent manner (38, 135–137).

## Abnormal energy metabolism in kidney-Yang deficiency

It was reported many years ago that pro-inflammatory stimuli inhibited Myc activity and cell proliferation, and participated in the enhancement of HIF-1α-dependent transcriptional programs and glycolysis (138). HIF-1α was induced in LPS-activated macrophages, which contribute to the induction of pro-inflammatory genes, especially IL-1β. The HIF-1α induction mechanism involved succinic acid, an intermediate product of the TAC with pro-inflammatory effects. HIF-1α also regulated pyruvate kinase M2 (PKM2) function (23). Metabolic reprogramming under inflammation is multifaceted. Sun et al. (25) summarized two main features of metabolic reprogramming of inflammation. First, it increased glycolysis and decreased lipid oxidative phosphorylation, which accelerated ATP production and biosynthesis; second, it showed epigenetic reprogramming to promote histone acetylation and inhibit DNA methylation, which is based on changes in the activity of acetyl and methyl donors and their metabolic enzymes. Fatty acid oxidation of myocardial mitochondria is the main source of ATP production. In the inflammatory state of heart failure, myocardial fatty acid uptake and mitochondrial oxidation are impaired, and energy metabolism is abnormal (Figure 1). The increased production of ATP by glycolysis and ketone oxidation was compensated to some extent (139). The changes in energy metabolism that lead to the reduction of the efficiency of the failing heart are due to the transcriptional changes of the key enzymes of the metabolic pathway and epigenetic changes (27). Na^+^/K^+^-ATPase and Ca^2+^-ATPase are important pumps to maintain the homeostasis of intracellular sodium and potassium levels and the excitability of cardiomyocytes, and they are also the main energy consumers of myocardium. A lot of evidence shows that glycolysis and Na^+^/K^+^-ATPase pumps are coupled in function, which provides ATP fuel for the pump (140, 141). As shown in Supplementary Table 6, the deficiency of kidney yang leads to disorders of energy metabolism. In animals with Kidney-Yang deficiency, the Na^+^/K^+^-ATPase and Ca^2+^-ATPase activities in the liver, skeletal muscle and myocardium were reduced (Supplementary Table 6), while the mitochondrial ATP production decreased and AMP increased in the liver and myocardium (Supplementary Table 6). The activities of SDH and respiratory chain complexes I, II, and III in the liver, skeletal muscle, and myocardium of kidney-Yang deficiency rat models also declined (Supplementary Table 6). The mitochondrial protein expressions and urine metabolite levels changed both in rat models and patients with kidney-Yang deficiency symptoms (Supplementary Table 6). A recent study showed that 27 metabolites involving amino acid, energy, ketone body, fatty acid, and methane metabolism changed significantly with the treatment of Yougui pill, a classic prescription for kidney-Yang deficiency (Supplementary Table 6).

## The mechanism of active ingredients regulating metabolic reprogramming in heart failure

**Figure 4:**
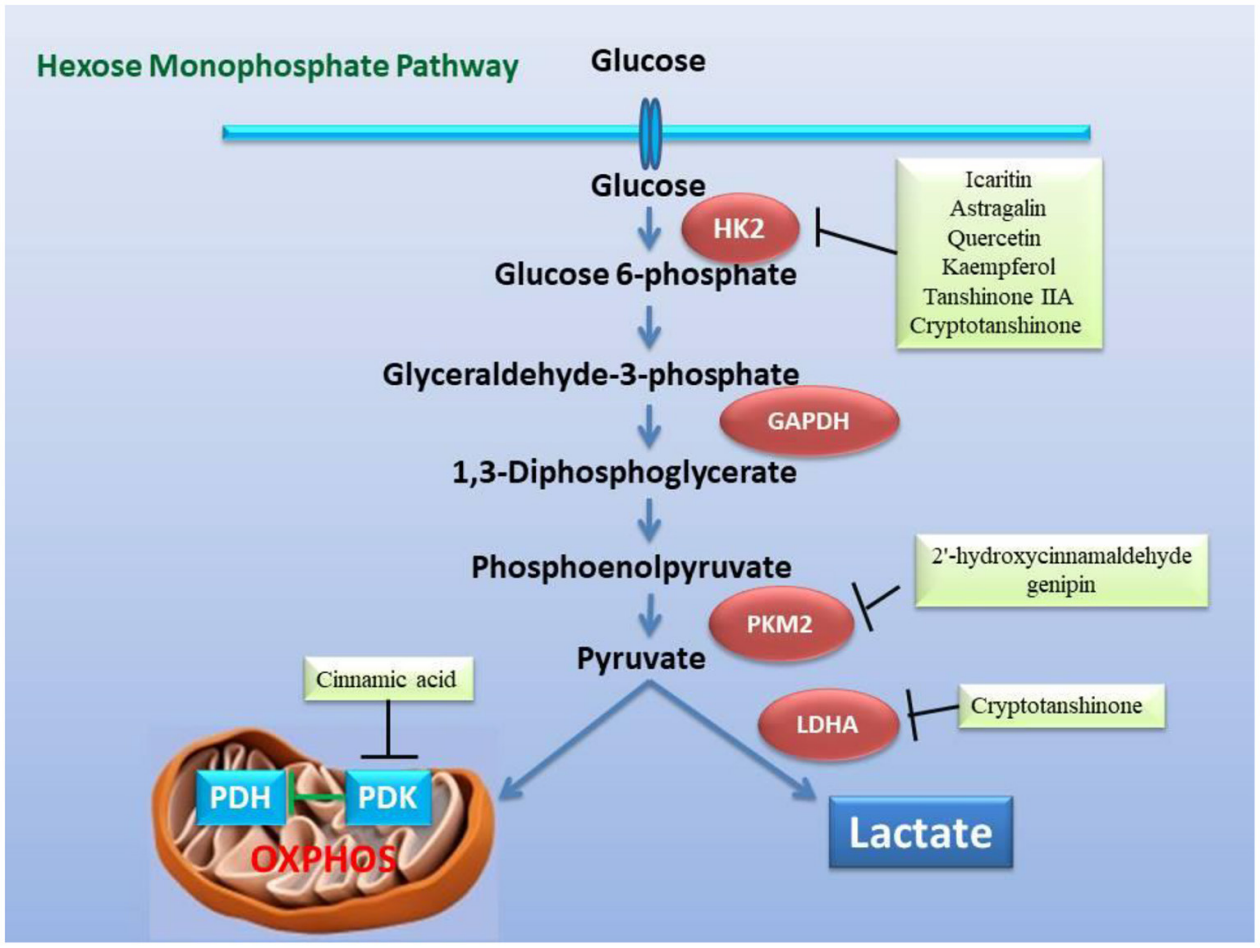
Inhibitory effect of the active ingredients of the classic formulas of invigorating kidney on the key enzymes related to glycolysis in the mitochondrial hexose pathway caused by heart failure. The active ingredients in the classic formulas have inhibitory effects on the enzymatic activity or expression of HK2, PKM2, LDHA, and PDK, respectively.

**Figure 5:**
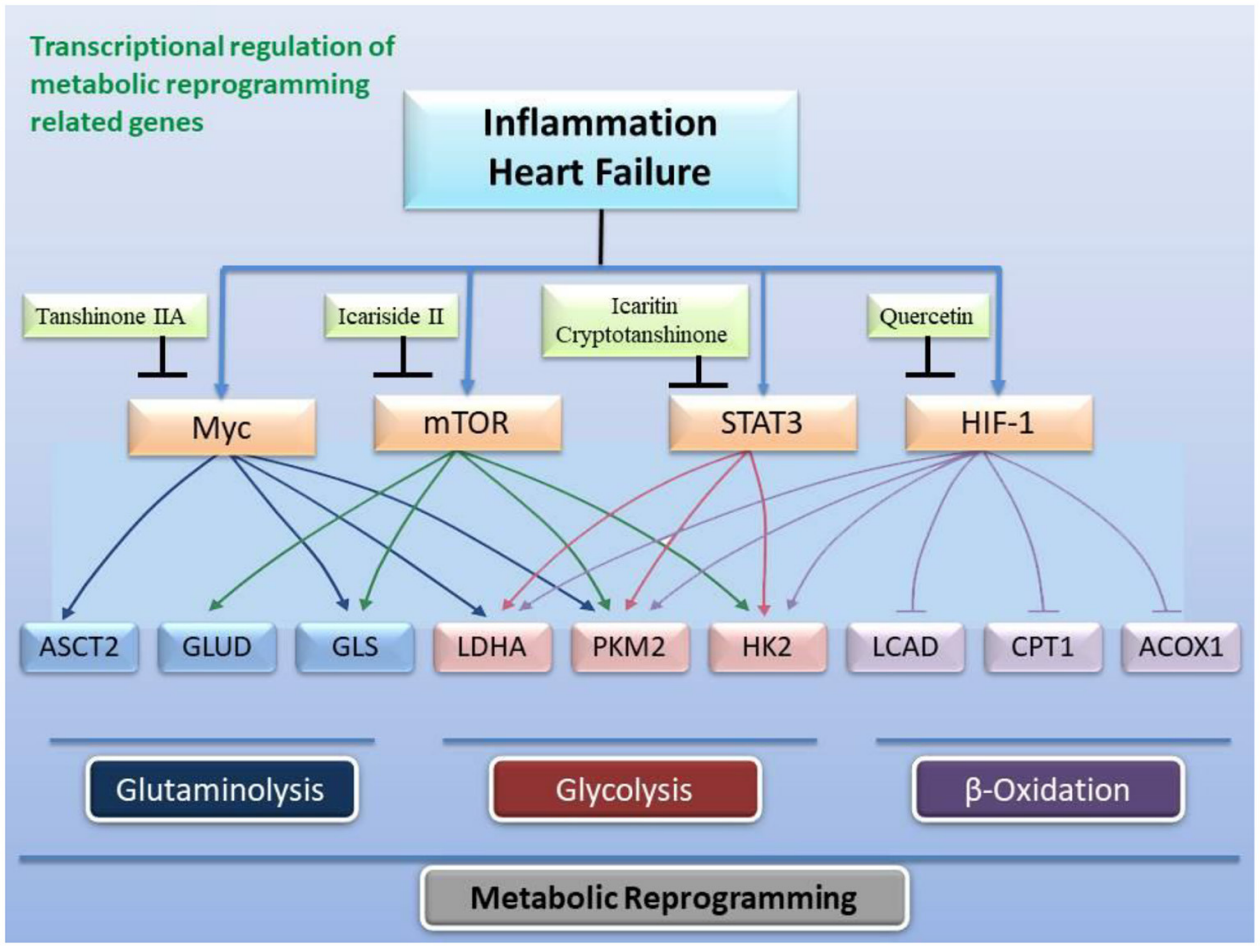
Transcriptional regulation of gene expression related to metabolic reprogramming by active ingredients in classical formulas. Heart failure and inflammation trigger the activity of transcription factors c-Myc, mTOR, STAT3, and HIF-1 to change the expression and activity of enzymes related to glycolysis, fatty acid β-oxidation, and glutaminolysis.

**Figure 6:**
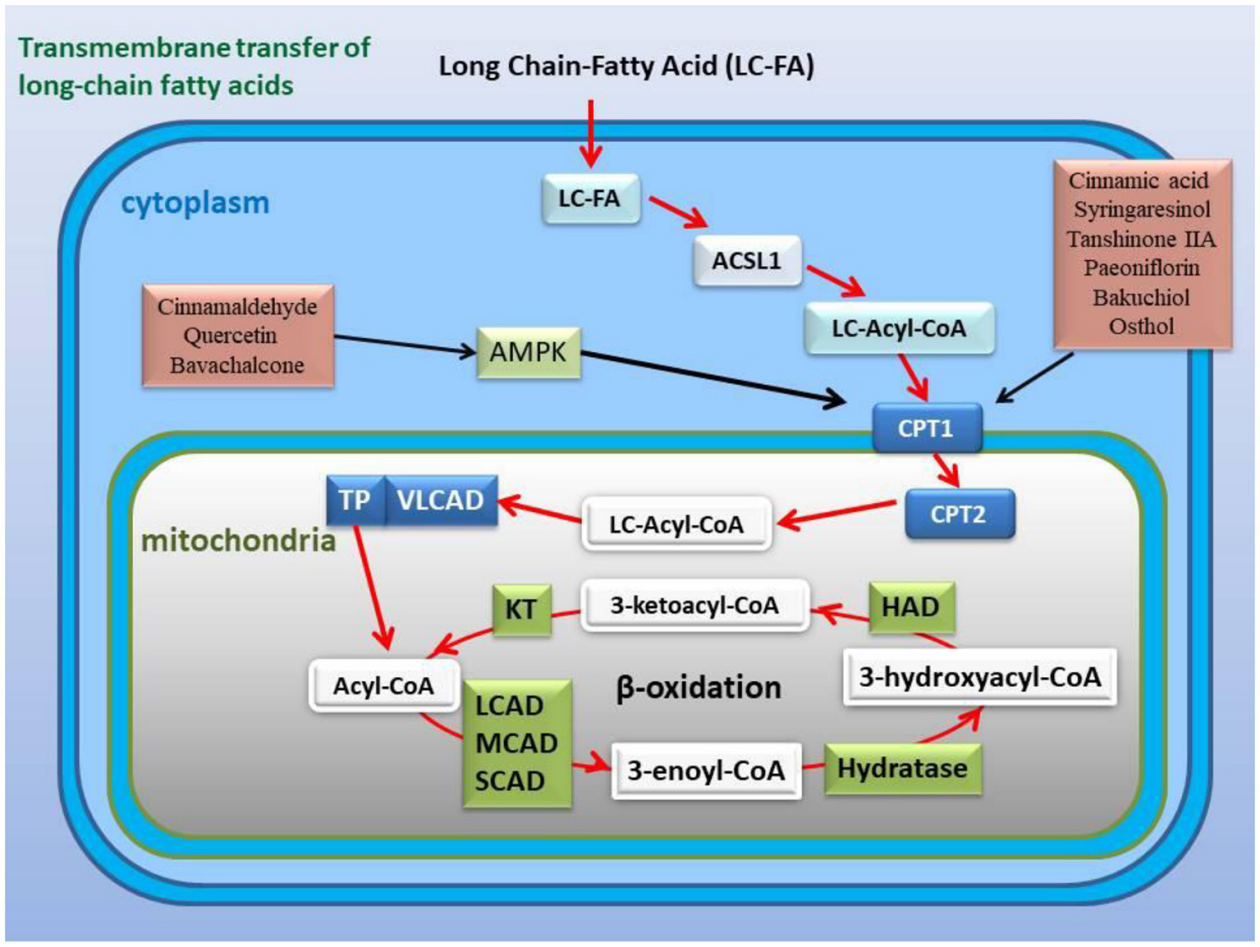
Active ingredients in the classic formulas activate the expression or activity of CPT1 and improve fatty acid β-oxidation with or without the AMPK pathway. CPT1 is a key channel for long-chain fatty acids across the mitochondrial outer membrane, and its activity or expression affects the β-oxidation of fatty acids.

**Figure 7:**
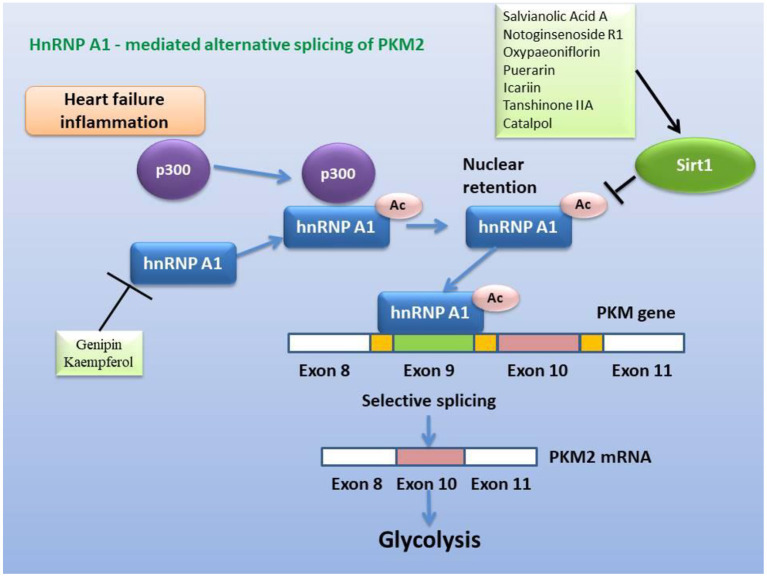
Inhibitory effect of active ingredients in classical recipes on hnRNP A1-mediated alternative splicing of PKM2, either directly or via activation of Sirt1. The heterogeneous nuclear ribonucleoprotein, which packs newly produced precursor mRNA, is acetylated and stabilized under conditions of heart failure and inflammation, and then selectively splices PKM2 mRNA to promote glycolysis.

Inflammation induces reprogramming of cellular energy production and biosynthesis to ensure faster ATP production and biosynthesis for defense response and damage repair by increasing glycolysis and reducing fatty acid β-oxidation and promoting mitochondrial glutaminolysis (25). Now, many studies have revealed that glycolysis promoted inflammation, and that inflammation and glycolysis formed an interdependent and mutually promoting relationship (142–144), and on the contrary, controlling glycolysis reversely regulated inflammation (145). Metabolic remodeling precedes occurrence of cardiac hypertrophy, and more importantly, metabolic remodeling promotes cardiac hypertrophy (145, 146). Changes in cardiac energy metabolism promoted the development of heart failure and increased the severity of heart failure (147, 148). In patients with heart failure, the protein expression of the TAC enzymes and the pyruvate dehydrogenase complex subunits of the heart are up-regulated, and the expression of the proteins related to fatty acid oxidation and oxidative phosphorylation complex are down-regulated, which indicated that the substantial metabolic switch from free fatty acid oxidation to glycolysis in heart failure (149, 150). Numerous studies have determined that hexokinase 2 (HK2) (151, 152), PKM2 (153, 154), and lactate dehydrogenase A (LDHA) (155, 156) are key genes involved in glycolysis (Figure 4). Moreover, glycolysis is regulated by transcription factors such as HIF-1α (23, 157), mTOR (158, 159), STAT3 (160, 161), c-Myc (162, 163) (Figure 5), and others. Alteration of mitochondrial fatty acid oxidation is another side of metabolic reprogramming. The change of heart fatty acid oxidation varies with the type of heart failure. Fatty acid oxidation of myocardium increases in diabetes and obesity related heart failure, and decreases in hypertensive heart failure related to ischemia. Earlier reports pointed out that the activity of the long-chain fatty acid transporter carnitine palmitoyltransferase I (CPT1) in the hypertrophic heart was lower than that in the heart of control animals (164). As the rate-limiting step of mitochondrial β-oxidation, the CPT1 deficiency exacerbates cardiac hypertrophy caused by pressure overload (165) Moreover, inhibition of CPT1 induced myocardial hypertrophy and premature death in mice (166). Specific expression of acyl-CoA synthase-1 (ACSL1) in the mouse heart promoted the synthesis of long-chain fatty acids (LCFA), contributed to the oxidation of LCFA, reduced the lipotoxicity caused by transverse aortic constriction stress, and delayed progressive heart remodeling and failure (167). Long-chain 3-hydroxyacyl-CoA dehydrogenase (LCAD) is a β-oxidation rate-limiting enzyme. In the hearts of LCAD deleted mice, acetyl carnitine was extremely insufficient, which resulted in severe cardiac energy deficiency, and the mice developed cardiac hypertrophy and decreased left ventricular ejection fraction and diastolic filling rate (168–170) (Figure 6). Studies have reported that the HIF-1 transcription factor, which is sensitive to hypoxia, inhibited the expression of CPT1 and LCAD, and controlled fatty acid metabolism (171, 172). Cardiac fibrosis is the core pathology of heart failure. Inflammation and specific pro-fibrotic factors, such as transforming growth factor-β, contribute to the accumulation of extracellular matrix in the heart and the proliferation of cardiac fibroblasts (173, 174). Glutaminolysis is a part of metabolic reprogramming and is required for tissue fibrosis (175, 176). The differentiation and activation of fibroblasts induced by TGF-β1 includes glutaminolysis (177). Inhibition of glutaminase 1, a key enzyme of glutaminolysis in fibroblasts, attenuates tissue fibrosis (178). In addition, many reports point out that glutaminolysis is the driver of pulmonary hypertension and cardiac remodeling (179, 180). c-Myc is believed to be involved in the development of heart failure (181, 182) and the regulation of glutaminolysis (183, 184). The basic pathology of inflammation and metabolic reprogramming exist in heart failure. In recent years, accumulated evidence has gradually established the knowledge that heart failure includes the underlying pathology of inflammation and the phenomenon of metabolic reprogramming, which provides new pharmacological targets (Figures 4–7).

In view of the fact that metabolic remodeling precedes and promotes the occurrence of myocardial hypertrophy, the inhibition of metabolic remodeling will contribute to the improvement of myocardial hypertrophy and heart failure. Animal and cell experiments have confirmed that the active ingredients of many herbs for “warming and tonifying Kidney-Yang” and “promoting blood circulation and dispersing blood stasis” from classic prescriptions have the effect of inhibiting metabolic reprogramming. *Cinnamomum cassia* is an important herbal medicine for warming and tonifying Kidney-Yang, whose active ingredients have the ability to inhibit glycolysis. Cinnamic acid blocks pyruvate dehydrogenase kinase activity (185), while 2'-hydroxycinnamaldehyde ameliorates PKM2-STAT3 signaling (186) (Figure 4). *Epimedium brevicornum* has been widely used in warming and nourishing kidney-yang for centuries. Its component icariside II acts as a natural inhibitor of mTOR to correct abnormal energy homeostasis (187), while icaritin inhibits IL-6-induced STAT3 pathway and HK2 expression (188) (Figure 5). Genipin, an active ingredient of *Eucommia ulmoides*, is also an inhibitor of UCP2. Studies have pointed out that genipin prevents the expression of glycolysis-related genes hnRNPA2/B1 and PKM2 (189, 190) (Figure 7). The active components of dodder seeds, such as astragalin, quercetin, and kaempferol, also play a role in reducing the expression of HK2 and improving glycolysis (191–193) (Figure 4). A recent study indicated that Kaempferol also reversed aerobic glycolysis by modulating hnRNPA1-PKM2 axis (194) (Figure 7). Besides, quercetin has also been found to stabilize HIF-1α, reduce the formation of heterodimers with β subunits (HIF-1β/ARNT), and inhibit aerobic glycolysis against glucose fluctuations (195, 196) (Figure 5). *Salvia miltiorrhaza* is an important herb for promoting blood circulation and removing blood stasis. Its main active components tanshinone IIA and cryptotanshinone have good anti-inflammatory effects (197–203). Tanshinone IIA inhibits HK2-mediated glycolysis, glucose consumption, and lactic acid production, blocks the effect of c-Myc conduction (200) (Figure 5). Cryptotanshinone down-regulates the expression of glycolysis-related genes, such as GLUT1, LDHA, and HK2, also cuts off STAT3-mediated glycolysis (201, 202) (Figure 5). Moreover, rosmarinic acid, is one of the components of *Salvia miltiorrhiza*, also has the effect of inhibiting glycolysis (203, 204). In addition, many other active ingredients of kidney-tonifying and blood-activating herbs, such as bergapten, baohuoside I, ligustilide, isoliquiritigenin, and curcumin, all reveal inhibitory effects on glycolysis (205–209) (Figure 4).

CPT-1 is a key restriction enzyme for LCFA β-oxidation. In inflammation and sepsis, the activity of myocardial CPT-1 is inhibited, which is a target of oxidative modification and a marker of myocardial dysfunction (210). Studies have shown that the active ingredients of *Ramulus Cinnamomi*, cinnamic acid, and syringaresinol, up-regulate the expression of CPT-1 through activating AMPK and PPARβ signaling (211, 212) (Figure 6). CPT-1 is a downstream gene regulated by AMPK pathway (213, 214). Activating blood herbal components, paeoniflorin from *Paeonia suffruticosa* and *Paeonia veitchii*, and tanshinone IIA from *Salvia miltiorrhiza*, restored the expression of CPT-1 through AMPK signaling pathway (215, 216) (Figure 6). Many components of kidney-tonifying herbs activate AMPK activity, such as cinnamaldehyde, quercetin, and bavachalcone (217–219), indicating that these active components may regulate fatty acids β-oxidation (Figure 6). The psoralen extract and the active ingredient bakuchiol, from kidney-tonifying herbal Psoraleae Fructus, reversed reverse the decline in the expression of PGC-1α and CPT-1 caused by cell senescence (220). Similar to the above examples, osthol, the active component of *Cnidium monnieri*, also has been reported to decrease SREBP-1c and increase CPT-1α expression (221) (Figure 6).

Metabolic reprogramming is a significant feature of failing hearts. As a biomarker of the Warburg effect, the activity of PKM2 is enhanced (222). While PKM2 maintains the aerobic glycolysis pathway, PKM2 also plays a key role in glutaminolysis (153, 223). Because the differentiation and activation of fibroblasts induced by TGF-β1 includes glutaminolysis (177), the prevention of glutaminolysis can contribute to antagonize cardiac fibrosis. However, there are few reports on glutaminolysis inhibitors, only tanshinone IIA and curcumin were found to down-regulate PKM2 expression (209, 224).

## Conclusion and expectations

Deficiency of heart- and kidney-Yang is the key pathogenesis of chronic heart failure, which reflects the more serious pathological phenotype of heart failure. According to NYHA's classification of heart failure, patients with kidney-Yang deficiency are mainly elderly and distributed in classes III + IV. Warming and invigorating kidney-Yang has become an important TCM therapy for chronic heart failure. Numerous classic and folk prescriptions embody the therapeutic cogitation of warming and tonifying kidney-Yang. An important modern pharmacological mechanism of this therapy is anti-inflammatory, neurohumoral regulation, and maintaining the homeostasis of cardiac function. Given that metabolic reprogramming is closely linked to inflammation, cardiac hypertrophy, and fibrosis, abnormal energy metabolism is a reflection of the inflammatory phenotype in chronic heart failure on metabolic reprogramming. The key components of classic tonifying-kidney prescriptions can regulate the energy metabolism abnormality of heart- and kidney-yang deficiency, and modulate the expression and activity of enzyme genes in glycolysis and fatty acid β-oxidation pathways. These discussions provide a modern pharmacological interpretation of the mechanism of tonifying-kidney therapy in the treatment of chronic heart failure.

At present, the design of clinical studies on chronic heart failure with classic prescriptions for kidney-tonifying lacks a multi-center, randomized, double-blind, placebo-controlled study based on standard treatment and parallel groups, and the production of classic prescriptions used lacks standardization, which requires major improvements. Besides, network pharmacology and protein target discovery of animal experiments on chronic heart failure with classic kidney-invigorating prescriptions and active ingredients need further improvements to discover and screen core targets, and finally fully elucidate the molecular mechanism of classic kidney-invigorating prescriptions on chronic heart failure treatment. Metabolic reprogramming is an important mechanism of chronic heart failure, which is linked to inflammation, cardiac hypertrophy, and fibrosis. Considering the different pathological mechanisms between HFpEF and HFrEF, it is of great clinical significance to study the classical formulations and their active components on the metabolic phenotypes, underlying molecular mechanisms, and potential therapeutic targets of fatty acid oxidation, glucose oxidation, and ketone body oxidation in chronic failing hearts.

## Author contributions

LC: conceptualization, data collection, and writing. DY: conceptualization and data collection. SL: study design, data collection, and writing. J-WX: study design, data collection, writing, and financial support. All authors read and approved the final manuscript.

## Funding

This work was supported by grants from the Specialized Research Fund for the National Natural Science Foundation of China (81973511).

## Conflict of interest

The authors declare that the research was conducted in the absence of any commercial or financial relationships that could be construed as a potential conflict of interest.

## Publisher's note

All claims expressed in this article are solely those of the authors and do not necessarily represent those of their affiliated organizations, or those of the publisher, the editors and the reviewers. Any product that may be evaluated in this article, or claim that may be made by its manufacturer, is not guaranteed or endorsed by the publisher.

## Glossary

6MWD: 6-min walking distance;

11β-HSD2: 11 beta-hydroxysteroid dehydrogenase-2;

AAT: anti-aging tablet;

ABC transporters: ATP-binding cassette transporters;

ACE: angiotensin-converting enzyme;

ACHE: acetylcholinesterase;

ACSL1: acyl-CoA synthase-1;

ADRA: adrenoceptor alpha subtype;

ADRBK1: androgenic, beta, receptor kinase 1;

AGTR1/AT1 receptor: angiotensin II receptor type 1;

AHR: aryl hydrocarbon receptor;

AKR1C3: aldo-keto reductase family 1 member C3;

ALD: aldosterone;

ALOX5: arachidonate 5-lipoxygenase;

AMP: adenosine monophosphate;

AMPK: AMP-activated protein kinase;

Ang II: angiotensin II;

ANP: atrial natriuretic peptide;

AQP-2: aquaporin 2;

AR: androgen receptor;

ARNT: aryl hydrocarbon receptor nuclear translocator;

ATP: adenosine triphosphate;

ATP1A1/2: ATPase Na^+^/K^+^ transporting subunit alpha 1;

AVP: arginine vasopressin;

BCL2: BCL2 apoptosis regulator/B-cell CLL/lymphoma 2;

BNP: brain natriuretic peptide;

Ca^2+^-ATPase: calcium pump in sarcoplasmic reticulum or plasma membrane;

CACNA1G/L: low-voltage-activated Ca (v)3.1 T-type calcium channel subtypes G/L;

CASP: caspase;

CAT: catalase;

CCE: composite cardiac event;

cGMP: cyclic guanosine monophosphate;

CHRM1: cholinergic receptor muscarinic 1;

CK-MB: creatine kinase-MB;

CI: cardiac index;

CO: cardiac output;

COX-2/ PTGS2: cyclooxygenase 2 or prostaglandin-endoperoxide synthase 2;

CPS: carbamyl phosphate synthase;

CPT1: carnitine palmitoyltransferase 1;

CRH: corticotropin-releasing hormone;

CSAR: cardiac sympathetic afferent reflex;

CYP: cytochrome P450;

DLD: dihydrolipoamide dehydrogenase;

E/A ratio: the ratio of the early (E) to late (A) ventricular filling velocities;

EF: ejection fraction;

EGFR: epidermal growth factor receptor;

eNOS/NOS3: endothelial nitric oxide synthase;

EPO: erythropoietin;

ERK1/2: extracellular signal-regulated kinases 1/2;

ESR1: estrogen receptor alpha;

FGF23: fibroblast growth factor 23;

FS: fractional shortening;

GLUT1: glucose transporter type 1;

GR: glutathione reductase;

GSTM1/2: glutathione S-transferase mu 1;

GSTP1: glutathione S-transferase pi 1;

GUCY1B3: guanylate cyclase 1 soluble subunit beta 3;

HFpEF: heart failure with reduced ejection fraction;

HIF-1α: hypoxia inducible factor-1alpha;

HK2: hexokinase 2;

HMGB1: high mobility group box 1;

hnRNPA2/B1: heterogeneous nuclear ribonucleoprotein A2/B1;

HO-1: heme oxygenase-1;

HR: heart rate;

hs-CRP: high-sensitivity C-reactive protein;

ICAM1: intercellular adhesion molecule 1;

ICR: the Institute of Cancer Research in the USA;

IFN-γ: interferon-gamma;

IKBKB: inhibitor of nuclear factor kappa B kinase subunit beta;

IL-1β: interleukin-1beta;

IL-6: interleukin-6;

iNOS/NOS2: inducible nitric oxide synthase;

JNK: c-Jun N-terminal kinase;

JSP: Jinkui Shenqi pill;

α-KGDH: alpha ketoglutarate dehydrogenase;

KM: Kunming;

LCAD: long-chain 3-hydroxyacyl-CoA dehydrogenase;

LCFA: long-chain fatty acids;

LDH: lactate dehydrogenase;

LOX-1: lectin-like oxidized low-density lipoprotein receptor-1;

LPS: lipopolysaccharides;

LVDD: left ventricular diastolic dysfunction;

LVEDd: left ventricle end diastolic dimension;

LVEDV: left ventricular end-diastolic volume;

LVEF: left ventricular ejection fraction;

LVESd: left ventricular end-systolic diameter;

LVESV: left ventricular end systolic volume;

LVSD: left ventricular systolic dysfunction;

MAPK: mitogen-activated protein kinase;

MCAD: acyl CoA dehydrogenase;

MCP-1: monocyte chemoattractant protein-1;

MLHFQ: Minnesota living with heart failure questionnaire;

MMP-2/9: matrix metallopeptidase 2/9;

MR: mineralocorticoid receptor;

mTOR: mammalian target of rapamycin;

Mϕ: macrophages;

Na^+^/K^+^-ATPase: sodium-potassium pump in outer plasma membrane;

NCOA1: nuclear receptor coactivator 1;

NE: norepinephrine;

NF-κB: nuclear factor kappa light chain enhancer of activated B cells;

NK cells: natural killer cells;

NLR: nucleotide-binding domain, leucine rich repeat containing receptor;

NLRP3: NLR family pyrin domain containing 3;

NO: nitric oxide;

NOX: nicotinamide adenine dinucleotide phosphate oxidase;

Nrf2: nuclear factor erythroid 2-related factor 2;

NT-proBNP: N-terminal pro-B-type natriuretic peptide;

NYHA: New York Heart Association;

ox-LDL: oxidized low-density lipoprotein;

OXPHOS: oxidative phosphorylation;

PaO_2_: partial pressure of oxygen;

PaCO_2_: partial pressure of carbon dioxide;

PDH: pyruvate dehydrogenase;

PDK: pyruvate dehydrogenase kinase;

PGC-1α: peroxisome proliferator-activated receptor-gamma coactivator-1alpha;

PGE2: prostaglandin E2;

PGR: progesterone receptor;

PI3K: phosphatidylinositol-3 kinase;

PKG: cyclic GMP-dependent protein kinase;

PKM2: pyruvate kinase M2;

PLAU: plasminogen activator, urokinase;

PON1: paraoxonase 1;

PPARs: peroxisome proliferator-activated receptors;

PRKCA: protein kinase C alpha;

PVN: paraventricular nucleus;

RCRI/Lee's score: revised cardiac risk index score;

RELA: RELA proto-oncogene, NF-kB subunit;

RVID: right ventricular internal diameter;

RVOT: right ventricular outflow tract;

SaO_2_: oxygen saturation;

SARDH: sarcosine dehydrogenase;

SV: stroke volume;

SD: Sprague-Dawley;

SDH: succinate dehydrogenase complex;

SELE: selectin E;

SFO: subfornical organ;

SI: stimulation index;

SLP: splenic lymphocyte proliferation;

SOD: superoxide dismutase;

Stat3: signal transducer and activator of transcription 3;

TAC: tricarboxylic acid cycle;

TC: therapeutic compound;

TCM: traditional Chinese medicine;

TGF-β1: transforming growth factor-beta1;

TLR: Toll-like receptor;

TNF-α: tumor necrosis factor-alpha;

TRPC6: canonical transient receptor potential channel 6;

UTR: urotensin II receptor;

VCAM1: vascular cell adhesion molecule 1;

VEGFA: vascular endothelial growth factor A;

WBC: white blood cell;

Yang-Qi: Yang energy.
