# Supplementary material for: Mechanism of tonifying-kidney Chinese herbal medicine in the treatment of chronic heart failure
Source: Front Cardiovasc Med. 2022 Sep 12;9:988360. doi: 10.3389/fcvm.2022.988360 (PMC9510640; doi:10.3389/fcvm.2022.988360)
Supplement: Supplementary file 1 [file Data_Sheet_1.docx]

**Supplementary tables**

**Supplementary** **Table 1 Clinical characteristics of several classical prescriptions for chronic heart failure**

| Classic prescriptions | Number of patients  (Treatment/control) | Course of treatment | Improvement of clinical  phenotype | Cardiac function | Blood parameters | Remarks | References |
| --- | --- | --- | --- | --- | --- | --- | --- |
| Zhenwu Decoction | 39/39 | 2 weeks | Therapeutic effects of TCM symptoms: treatment (89.74%) > control (64.10%) | LVEDd↓, LVESd↓, LVEF↑, E/A↑ | NT-proBNP↓, hs-CRP↓ | Zhenwu decoction in the treatment of chronic heart failure on the basis of conventional Western medicine | [1-3] |
|  | 40/23 | 2 weeks | Therapeutic effects of TCM symptoms: treatment (82.5%)> control (56.0%) | LVEF↑, LVSD↓, LVDD↓ | BNP↓, hs-CRP↓ |  |  |
|  | 60/60 | 2 weeks | Therapeutic effects of TCM symptoms: treatment(93.33%) > control (80.0%); palpitation↓, dyspnea↓, edema↓, cold chills limbs↓ | LVEF↑ | NT-proBNP↓,Serum K↓ |  |  |
| Sini decoction | 28/28 | 4 weeks | Therapeutic effects of TCM symptoms: treatment (85.71%) > control (67.86%) | RCRI/Lee's score↓ | NT-proBNP↓ | Sini decoction in the treatment of chronic heart failure on the basis of conventional Western medicine | [4-6] |
|  | 52/52 | 4 weeks | Therapeutic effects of TCM symptoms: treatment (score:5.42±0.85) < control (score:20.08±1.95) | RCRI/Lee's score↓, 6MWD↑, LVEF↑, SV↑ | NT-proBNP↓, cTnI↓, CK-MB↓, hs-CRP↓ |  |  |
|  | 89/89 | 4 weeks | Total effective rate of NYHA functional classification: treatment (91.01%) > control (78.65%) | RCRI/Lee's score↓, 6MWD↑ | NT-proBNP↓ |  |  |
| Lingguizhugan decoction | 90/30 | 3 months | Therapeutic effects of TCM symptoms: treatment (73.33%)> control (60.00%)  TCM syndrome score: treatment (score:5.79±1.43) < control (score:7.26±2.41) | LVSD↓, LVDD↓, LVEF↑ | ANP↓ | Lingguizhugan decoction in the treatment of chronic heart failure on the basis of conventional Western medicine | [7,8] |
|  | 37/37 | 4 weeks |  | LVEF↑, LVEDd↓, LVESd↓ | NT-proBNP↓,sST2↓ |  |  |
| Shenfu injection | 70/69 | 7±1 days | Total effective rate of NYHA functional classification: treatment (78.38%) > control (61.43%); therapeutic effects of TCM symptoms: treatment (89.19%) > control (60.00%) | Effective rate of RCRI/Lee's score: treatment (70.27%) > control (52.17%), CCE↓, 6MWD↑, LVEF↑, | -- | Shenfu injection in the treatment of chronic heart failure on the basis of conventional Western medicine | [9-11] |
|  | 39/45 | 2 weeks | Therapeutic effects of TCM symptoms: treatment (89.74%) > control (71.11%); palpitation↓, cold chills limbs↓, shortness of breath and tiredness↓ | SaO_2_↑, PaO_2_↑, PaCO_2_↓,RVID, RVOT, LVEF↑ | NT-proBNP↓,ET-1↓ |  |  |
|  | 33/32 | 10 days | Incidence rate of heart failure: treatment (27.27%) < control (53.12%), | RCRI/Lee's score↓, 6MWD↑, LVEF↑, | BNP↓, hs-CRP↓, CysC↓ |  |  |
| Bushen Huoxue Decoction | 54/52 | 4 weeks | Therapeutic effects of TCM symptoms: treatment(94.4%) > control(63.5%) | LVEF↑, LVESd↓, LVESV↓,SV↑, | BNP↓ | Bushen Huoxue decoction in the treatment of chronic heart failure on the basis of conventional Western medicine | [12, 13] |
|  | 105/105 | 4 weeks | Therapeutic effects of TCM symptoms: treatment(95.24%) > control (81.90%) | 6MWD↑, LVEF↑, HR↓, SV↑, CO↑, CI↑, | hs-CRP↓ |  |  |
| Qili Qiangxin Capsule | 256/256 | 12 weeks | NYHA III/IV: treatment (15.21%) < control (26.98%) | 6MWD↑, CCE↓, LVEF↑,MLHFQ↑ | NT-proBNP↓ | Qili Qiangxin capsules in the treatment of chronic heart failure on the basis of conventional Western medicine | [14,15] |
|  | 54/54 | 4 weeks | Total effective rate: treatment (90.74%) > control (72.22%) | 6MWT↑, LVEF↑, LVESV↓, LVEDV↓, | NT-proBNP↓, cTnI↓ |  |  |

6MWD: 6-min walking distance; ANP: Atrial natriuretic peptide; CCEs:Composite cardiac events; CK-MB: Creatine kinase-MB; CI: Cardiac index; CO: Cardiac output; BNP: brain natriuretic peptide; E/EG HR: heart rate ; hs-CRP: high-sensitivity C-reactive protein; LVDD: Left ventricular diastolic dysfunction; LVEDd: Left Ventricle End Diastolic Dimension; LVEDV: Left ventricular end-diastolic volume ; LVEF: left ventricular ejection fraction; LVESd: left ventricular end-systolic diameter; LVESV: left ventricular end systolic volume ; LVSD: Left ventricular systolic dysfunction; MLHFQ: Minnesota Living With Heart Failure Questionnaire; NT-proBNP: N-terminal pro-B-type natriuretic peptide; PaO_2_: partial pressure of oxygen; PaCO_2_: partial pressure of carbon dioxide; RVID: right ventricular internal diameter; RVOT: right ventricular outflow tract; SaO_2_: oxygen saturation; SV: stroke volume; NYHA: New York Heart Association; RCRI/Lee's score: revised cardiac risk index score

**References in supplementary tables**

1. Zou Y, Li W. Clinical observation on Zhenwu Decoction with chemical medicine for congestive heart failure (Article in Chinese). Journal of Liaoning University of Traditional Chinese Medicine (2016) 18:79-81.
2. Zhao J, Li D, Guan Y, Zhu F, Wu F, Xu X. Clinical Observation on Effect of Modified Zhenwu Decoction in Patients with Chronic Heart Failure and Yang Deficiency of Heart-Spleen-Kidney and Blood Stasis (Article in Chinese). China Journal of Traditional Chinese Medicine and Pharmacy (2015)33:2915-2917.
3. Yu J, Zhang X, Liu Y, Wu C, Li L, Liang T, Liang J, Bi W. Clinical Study of Zhenwu Decoction on Acute Decompensated Heart Failure Patients with Diuretic Resistance (Article in Chinese). Journal of Emergency in Traditional Chinese Medicine (2020)29:79-82.
4. Jiang Z. Clinical efficacy of the Sini decoction on chronic heart failure (Article in Chinese). Clinical Journal of Chinese Medicine (2018)10:27-29.
5. Su H, Wu H, Li C. Clinical research of senile chronic heart failure treated with the modified Sini decoction (Article in Chinese).World Journal of Integrated Traditional and Western Medicine (2019)14:974-977.
6. Yin J, Gong Y, Gu W. Clinical effect of Jiawei Fuling Sini Decoction in the treatment of chronic heart failure (Article in Chinese). China Medical Herald (2020)17:151-155.
7. Li X, Geng X, Wang S, Jiang H, Lai Z. Clinical study on Linggui Zhugan Decoction in treating congestive heart failure (Article in Chinese).China Journal of Traditional Chinese Medicine and Pharmacy(2005) 20:220-222.
8. Shi Z, Zhang Y. Clinical study on Linggui Zhugan Decoction for chronic heart failure (Article in Chinese). Journal of New Chinese Medicine (2021) 53:31-35.
9. Wang X, Zhao Z, Mao J, Du T, Chen Y, Xu H, Liu N, Wang X, Wu J, Li R, Xu Y, Zhao Y, Wang L, He J, Zhang J, Zhai J, Zhao G, Hou Y, Wang S, Liu C. Randomized, Double-Blinded, Multicenter, Placebo-Controlled Trial of Shenfu Injection for Treatment of Patients with Chronic Heart Failure during the Acute Phase of Symptom Aggravation (Yang and Qi Deficiency Syndrome). Evid Based Complement Alternat Med (2019) 2019:9297163.
10. Wang R, Lin D, Zhuo J, Lin T. Clinical Study of Shenfu Injection in the Treatment of Chronic Pulmonary Heart Disease Complicated with Heart Failure (Heart-Kidney Yang Deficiency Syndrome) (Article in Chinese). Journal of Emergency in Traditional Chinese Medicine (2022) 31:262-265.
11. Zhang Y, Cheng R, Shang Y, Qu X, Wang T, Lv X, Wang Q, Jiang H. Clinical observation of Shenfu Injection in treating acute myocardial infarction with heart failure (Article in Chinese). Journal of Emergency in Traditional Chinese Medicine (2019) 28:317-319.
12. Li J, Tang M, Song Y. Clinical observation of chronic heart failure treated with Bushen Huoxue Decoction (Article in Chinese). World Journal of Integrated Traditional and Western Medicine,2012,7(04):314-316.
13. Wang H, Kong L, Zhang R, Li Z, Xie C. Clinical study of Bushen Huoxue Decoction on chronic congestive heart failure (Article in Chinese). Chinese Journal of Integrative Medicine on Cardio-/Cerebrovascular Disease, 2016,14(08):866-868.
14. Li X, Zhang J, Huang J, Ma A, Yang J, Li W, Wu Z, Yao C, Zhang Y, Yao W, Zhang B, Gao R. Efficacy and Safety of Qili Qiangxin Capsules for Chronic Heart Failure Study Group. A multicenter, randomized, double-blind, parallel-group, placebo-controlled study of the effects of qili qiangxin capsules in patients with chronic heart failure. J Am Coll Cardiol. 2013 Sep 17;62(12):1065-1072.
15. Yao J, Liu D. Clinical Effect of Qili Qiangxin Capsule Combined with Sacubitril-Valsartan in Patients with Chronic Heart Failure. J Healthc Eng. 2022 Mar 23;2022:8598806.

**Supplementary table 2 Immune changes of Kidney-Yang deficiency in animal models**

| Study | Animals | Immune changes in Kidney-Yang deficiency (Compared with the control group) | | | | | | | | | | | | | | | | | | Therapy | |
| --- | --- | --- | --- | --- | --- | --- | --- | --- | --- | --- | --- | --- | --- | --- | --- | --- | --- | --- | --- | --- | --- |
|  |  | Thymus index | Spleen index | WBC count | CD4+ | CD8+ | CD4+/ CD8+ | T lymph IS | B lymph IS | NK activity | Mφ Phagocytosis | | IgG | IgM | SLP test | IL-2 | IL-4 | IFN-γ | IL-4/ IFN-γ | TC | Duration |
|  |  |  |  |  |  |  |  |  |  |  | Rate | Index |  |  |  |  |  |  |  |  |  |
| Lu et al. [16] | KM mice | **↓** | **↓** |  |  |  |  | **↓** | **↓** | **↓** |  |  |  |  |  |  |  |  |  | AAT | 4 w |
| Xu et al. [17] | SD rats | **↓** | **↓** | **↓** | **↓** | **↑** | **↓** |  |  |  | **↓** | **↓** | **↑** | **↑** |  |  |  |  |  | JSP | 30d |
| Zhao et al. [18] | SD rats |  |  |  | **↓** | **↑** | **↓** |  |  | **↓** |  |  |  |  |  |  |  |  |  | Yougui capsule | 28d |
| Li et al. [19] | SD rats | **↓** | **↓** |  |  |  |  |  |  |  | **↓** | **↓** |  |  | **↓** |  |  |  |  | Yougui pill | 28d |
| Zhang et al. [20] | ICR male mice |  |  |  |  |  |  |  |  | **↓** |  |  |  |  | **↓** | **↓** |  | **↓** |  | --- | --- |
| Yan et al. [21] | male mice | **↓** | **↓** |  |  |  |  |  |  |  |  |  |  |  |  |  | **↑** | **↓** | **↑** | JSP | 6w |

AAT: Anti-aging Tablet; JSP: Jinkui Shenqi Pill; KM: Kunming; Mφ: macrophages; SI: Stimulation index; SLP: Splenic lymphocyte proliferation; TC: Therapeutic compound; WBC: White blood cell

**References in supplementary tables**

1. Lu T，Pan Y，Leng X，Chen M，Shou Q，Zhou W，Tao T，Zhu K. Effects of Anti-aging Tablets on immune function of experimental mice models with deficiency of the kidney-yang (Article in Chinese). Journal of Gansu College of Traditional Chinese Medicine (2011) 28: 9-11.
2. Xu H, Yang S, Li S, Li T, Xu X, Li X. Effect of alcohol extract of Cuscuta chinensis on immune function in rats with kidney yang deficiency syndrome (Article in Chinese). Journal of Chinese Medicinal Materials (2015) 38:2163-2165.
3. Zhao M, Zhou A, Xu A, Cao J, Chen H. Effects of Yougui capsule on immune function of Kidney-Yang deficiency rats (Article in Chinese). Journal of Hubei University of Chinese Medicine (2013) 15: 18-20.
4. Li R, Jia H, Li X, Luo S. Immune effects of Kangshuailing Gao on rats with Kidney-Yang deficiency (Article in Chinese).Asia-Pacific Traditional Medicine (2018) 14: 14-16.
5. Zhang J, Shen X, Zhang H, Zhou Y. Changes of immune function in mice with Kidney-Yang deficiency (Article in Chinese). Zhejiang Journal of Traditional Chinese Medicine (2000) 35: 32.
6. Yan T, Li Z. Effect of Jingui Shenqi Pill on Th1/Th2 immunoregulation in mice with Kidney-Yang deficiency induced "Overwork, untidy sex" (Article in Chinese). Shandong Medical Journal (2010) 50: 29-30.

**Supplementary table 3 Network Pharmacological Analysis of Zhenwu Decoction in the treatment of chronic heart failure**

| Study | Analysis Tools | Signaling Pathways | Key Target Genes | Key Active Ingredients |
| --- | --- | --- | --- | --- |
| Wu et al. [22] | Databases: BATMAN-TCM, David 6. Softwares: Metascape | MAPK signaling, Adrenergic signaling in cardiomyocytes, Renin secretion, Synthesis and secretion of aldosterone, Renin-angiotensin system, Bicarbonate absorption in proximal tubules, Aldosterone regulates sodium reabsorption, cGMP PKG signaling, Calcium signaling | ACE, ADRA2A, ADRA2B, ADRBK1, AGTR1, ATP1A1, ATP1A2, CACNA1G, CACNA1L, GUCY1B3, TNF | N/A |
| Xu et al. [23] | Databases: TCMSP, GeneCards, UniPro, CTD, TTD, SwissTarget Prediction, Network Analyzer  Softwares: Cytoscape 3.2.1, RStudio, AutoDock Vina1.1.2, AutoDock Tools 1.5.6, PDB, PyMol 2.3.0, Discovery studio 2016, Chimera 1.13 | cGMP-PKG signaling, Renin secretion, Calcium signaling, cAMP signaling, Vascular smooth muscle contraction, Insulin resistance, Platelet activation, Arachidonic acid metabolism, Adrenergic signaling in cardiomyocytes, PI3K/Akt signaling | IL-6, NOS3, HMOX1, PTGS2, CAT, PPARG, TNF, VEGFA | Norcoclaurine, 3β-acetoxyatractylone, Kaempferol, Paeoniflorin, Stigmasterol, β-sitosterol |
| Ouyang et al. [24] | Databases: TCMSP, GeneCards, UniPro, STRING, Bioconductor  Softwares: Cytoscape 3.6.1, Active Perl, R3.6.1, VennDiagram. | Fluid shear stress and atherosclerosis, TNF signaling, Apoptosis, IL-17 signaling, NF-кB signaling, Cholinergic synapse, p53 signaling | IL-6,MAPK8, CASP3,RELA, ESR1, AR, AHR, CYP3A4, CYP1A1, PGR, ICAM1, PPARG, GSTP1, CASP8, CYP1B1, NCOA1, VCAM1, AKR1C3, CASP9, CHRM1, GSTM1, IKBKB, PON1, ACHE, ALOX5, BCL2, GSTM2, PLAU, PRKCA, SELE | β-sitosterol, stigmasterol, ergosterol, ergosterol endoperoxide, cerevisterol, paeoniflorin, paeoniflorgenin, kaempferol, dehydroandrographolide, hederagenin, (+)-catechin, karanjin, dehydrotrametenolic acid |
| Zhang et al. [25] | Databases: TCMSP, UniPro, GeneCards, STRING, DAVID 6.8  Softwares: Cytoscape 3.7.1, Venny 2.0.2 | Apoptosis, P53 signaling, NF- кB signaling, PI3K/Akt signaling, TLR signaling, TNF signaling, VEGF signal, IL-17 signaling, Adrenergic signaling in cardiomyocytes, HIF-1 signaling, Calcium signaling | IL-6, CASP3, ESR1, CASP8, CASP9, ACHE, BCL2, PLAU, PON1, ADRA1A | β-sitosterol, Stigmasterol, Deltoin, 3β-acetoxyatractylone, Norcoclaurine |
| Gao et al. [26] | Databases: TCMSP, KTKP, PubChem, Swiss Target Prediction, STITCH, UniPro, TTD, OMIM, DisGeNET, GeneCards, STRING, DAVID, PDB, systemsDock,  Softwares: Cytoscape 3.2.1, | Insulin resistance, Estrogen signaling, ABC transporters, PPAR signaling, Adherens junction, IL-17 signaling, PI3K-Akt signaling, p53 signaling, Apelin signaling, NF- кB signaling | PTGS2, EGFR, ESR1, NOS3, PPARG, MMP9, AHR, AR, CYP19A1 | Kaempferol, (+)-catechin, pentagalloylglucose, β-sitosterol, Stellasterol, mairin, hederagenin, trametenolic acid, 11,14-eicosadienoic acid, Norcoclaurine, 6-gingerol (24S)-24-Propylcholesta-5-ene-3beta-ol, |

**References in supplementary tables**

1. Wu Y, Zheng J, Zhu Z, Luo W. Mechanism of Zhenwu Decoction for Treatment of Chronic Heart Failure Based on Network Pharmacology (Article in Chinese). Journal of Guangzhou University of Traditional Chinese Medicine (2019) 36:1617-1623.
2. Xu H, Qi X, Fang C, Deng J, Shi P, Mo J, Liang H, Yuan T, Wu H. Study on the Mechanism of Zhenwu Decoction in Treating Chronic Heart Failure Based on Network Pharmacology (Article in Chinese). Chinese Journal of Modern Applied Pharmacy (2020) 37 :1802-1811.
3. Ouyang Y, Fu W, Huang C, He Y, Zhao X, Wang Q. Study on the Mechanism of Zhenwu Decoction in Treating Chronic Heart Failure Based on Network Pharmacology (Article in Chinese). Journal of Liaoning University of Traditional Chinese Medicine (2020) 88: 123-129.
4. Zhang L，Qiu Y，Bai Q，Gao J. Action Mechanism of Zhenwu Decoction in Preventing and Treating Chronic Heart Failure from Perspective of Network Pharmacology (Article in Chinese). Acta Chinese Medicine (2020) 35: 1763-1769.
5. Gao P, Zhang S, Jiao J, Lou N, Li X, Wang Y. Network Pharmacology-based Prediction of Potential Mechanism of Zhenwu Decoction Acting on Chronic Heart Failure. Study on the Mechanism of Zhenwu Decoction in Treating Chronic Heart Failure Based on Network Pharmacology (Article in Chinese). Journal of Basic Chinese Medicine (2021) 27: 132-139.

**Supplementary table 4**  **Main active ingredients** **of herbs for Kidney-tonifying and promoting blood circulation**

| Chinese name | Latin name | Medicinal parts | Major effective active ingredients [27] |
| --- | --- | --- | --- |
| Fuzi | *Aconitum carmichaelii* Debx | Dried roots | Norcoclaurine/Higenamine, Monkshood Polysaccharide, |
| Guizhi | *Cinnamomum cassia* Presl. | Dried twigs | Cinnamaldehyde, Cinnamic acid; Rosavin; Patchoulol; syringaresinol |
| Shanzhuyu | *Cornus officinalis* Sieb. et Zucc. | Dried ripe pulps | Cornuside, morroniside, Loganin; Sweroside |
| Duzhong | *Eucommia ulmoides* Oliver. | Dried barks | Syringaresinol; Aucubin; Genipin; Geniposide; Geniposidic acid; Betulin |
| Roucongrong | *Cistanche deserticola* Ma. | Dried fleshy stems | Acteoside, Echinacoside, Cistanoside A |
| Tusizi | *Cuscuta chinensis* Lam. | Dried mature seeds | Quercetin; astragalin; hyperin; kaempferol; Isorhamnetin |
| Buguzhi | *Psoralea corylifolia* Linn. | Processed fruits | Bakuchiol, Bavachin, Bavachalcone, bavachinin, Isobavachalcone, Bavachromene, Neobavachalcone, Bakuchalcone, Neobavaisoflavone |
| Yinyanghuo | *Epimedium brevicornum* Maxim. | Dried Stem and leaf | Icariin, icarisides, epimedins, baohuosides |
| Bajitian | *Morinda officinalis* How. | Dried roots | Monotropein, asperuloside, rubiadin, physcion |
| Shechuangzi | *Cnidium monnieri* (L.) Cuss | Dried Fruits | Osthole, bergapten, columbianadin, Imperatorin |
| Suoyang | *Cynomorium songaricum* Rupr. | Dried stems | Cynoterpene, acetylursolic acid, Catechin, phlorizin, Procyanidins |
| Niuxi | *Achyranthes bidentata* Blume. | Dried roots | Ecdysterone, inokosterone, Oleanolic acid |
| Honghua | *Carthamus tinctorius* Linn. | Dried flowers | Carthamin; safflower yellow A and B; dihydroactinidiolide |
| Danggui | *Angelica sinensis* (Oliv.)Diels. | Dried roots | Butylidenephthalide; Ligustilide; Scopoletin |
| Danshen | *Salvia miltiorrhiza* Bge*.* | Dried roots | Tanshinone IIA, cryptotanshinone , Salvianic acid A, rosmarinic acid, |
| Mudanpi | *Paeonia suffruticosa* Andr. | Root bark | Paeoniflorin, oxypaeoniflorin, paeonol |
| Chishao | *Paeonia veitchii* Lynch. | Dried roots | Paeoniflorin, oxypaeoniflorin, albiflorin, galloylpaeoniflorin, paeonol |
| Chuanxiong | *Ligusticum chuanxiong* Hort. | Dried roots | Tetramethylpyrazine, perlolyrine, ligustilide, senkyunolides, |
| Sanqi | *Panax notoginseng* (Burk.) F. H. Chen | Dried roots | Ginsenosides, gypenosides, notoginsenosides, dencichine |
| Yujin | *Curcuma wenyujin* Y. H. Chen et C. Ling | Dried roots | Curcumin, demethoxycurcumin, turmerone, germacrone, curdiene, cineole |
| Taoren | *Prunus persica* (L.) Batsch. | Dried mature seeds | amygdalin, 3-caffeoylquinic acid, prunasin |
| Jixueteng | *Spatholobus suberectus* Dunn | Dried cane | formononetin, prunetin, daidzein, epicatechin, isoliquiritigenin, |

**References in supplementary tables**

1. Editorial Board of Chinese Materia Medica organized by the State Administration of Traditional Chinese Medicine. Chinese Materia Medica (Book in Chinese). Shanghai: Shanghai Science and Technology Press (1999). 533p.

**Supplementary table 5 Formulas of Qili Qiangxin Capsule and Bushen Ningxin Granule, and their main active ingredients**

| Chinese name | | Latin name | Medicinal parts | Major effective active ingredients |
| --- | --- | --- | --- | --- |
| Qili Qiangxin Capsule | Fuzi | *Aconitum carmichaelii* Debx | Dried roots | Norcoclaurine/Higenamine, Monkshood Polysaccharide, |
|  | Huangqi | *Astragalus membranaceus* (Fisch.) *Bge.var. mongholicus* (Bge.) Hsiao | Dried roots | Calycosin, Astragaloside IV, |
|  | Guizhi | *Cinnamomum cassia* Presl. | Dried twigs | Cinnamaldehyde, Cinnamic acid; Rosavin; Patchoulol; syringaresinol |
|  | Yuzhu | *Polygonatum odoratum* (Mill.) Druce | Dried roots | homoisoflavanone‑1, |
|  | Honghua | *Carthamus tinctorius* Linn. | Dried flowers | Carthamin; safflower yellow A and B; dihydroactinidiolide |
|  | Renshen | *Panax ginseng* C.A.Mey. | Dried roots | ginsenosides, panaxatriol, 20-epiproto panaxadiol, 20(S)-protopanaxadiol |
|  | Zexie | *Alisma orientalis* (Sam.) Juzep. | Dried roots | alisol A , alisol A 24-Acetate, alisol B 23-Acetate, alismol |
|  | Danshen | *Salvia miltiorrhiza* Bge. | Dried roots | Tanshinone IIA, cryptotanshinone , Salvianic acid A, rosmarinic acid, |
|  | Tinglizi | *Lepidium apetalum* Willd. or *Descurainia sophia* (L.)Webb ex prantl | Dried mature seeds | sinapic acid, strophanthidin, sinapine, |
|  | Xiangjiapi | *Periploca sepium* Bge. | Dried root barks | periplocin, periplocymarin, |
|  | Chenpi | *Citrus reticulata* Blanco. | Dried fruit peels | hesperidin, naringin, poncirin |
| Bushen Ningxin Granule | Niuxi | *Achyranthes bidentata* Blume. | Dried roots | Ecdysterone, inokosterone, Oleanolic acid |
|  | Danggui | *Angelica sinensis* (Oliv.)Diels. | Dried roots | Butylidenephthalide; Ligustilide; Scopoletin |
|  | Danshen | *Salvia miltiorrhiza* Bge*.* | Dried roots | Tanshinone IIA, cryptotanshinone , Salvianic acid A, rosmarinic acid, |
|  | Chishao | *Paeonia veitchii* Lynch. | Dried roots | Paeoniflorin, oxypaeoniflorin, albiflorin, galloylpaeoniflorin, paeonol |
|  | Dihuang | *Rehmannia glutinosa* Libosch. | Dried roots | Leonuride, aucubin,catalpol, melittoside, rehmanniosides, melittoside, geniposide, 8-epiloganic acid |
|  | Cangshu | *Atractylodes Lancea* (Thunb.) DC. | Dried roots | β-eudesmol, atractylenolide |
|  | Boziren | *Platycladus orientalis* (L.)Franco | Dried seed kernel | Cedrol, pinusolide |
|  | Xiangfu | *Cyperus rotundus* L. | Dried rhizome | Isocyperol,Cyperol |
|  | Shíchangpu | *Acorus tatarinowii* Schott | Dried roots | Asarone, |
|  | Zelan | *Lycopus lucidus* Turcz.vat.hirtus Regel | Dried stem and leaf | Eupatilin, Rosmarinic acid, Lycopic acid A/B/C |
|  | Shanzha | *Crataegus pinnatifida* Bge. | Roasted to a burnt Brown fruits | Quercetin, Hyperin, Crataegolic acid, Amygdalin |
|  | Fuling | *Poria cocos* (Schw.) Wolf | Core part of the dried fungus | Eburicoic acid, Pachymic acid, Poricoic acids |
|  | Yimucao | *Leonurus japonicus* Houtt. | Dried grass | Leonurine, Stachydrine, Hispanolone |

**Supplementary table 6 Energy Metabolism Disorder of Kidney-Yang Deficiency**

| Parameters | Animols/  Human | Samples | Phenotype of kidney-Yang deficiency | References |
| --- | --- | --- | --- | --- |
| ATPase | Rats | Liver, Skeletal muscle, Myocardium | Na+/K+-ATPase↓, Ca2+/Mg2+-ATPase↓ | 28-32 |
| SDH | Rats | Liver, Skeletal muscle, Myocardium | SDH↓ | 30,31,33 |
| Adenosine Phosphate | Rats | Liver, Myocardium | Mitochondrial ATP↓, AMP↑ | 32, 33 |
| Respiratory chain complex activity | Rats | Liver | Complex I↓, Complex II↓, Complex III↓ | 32 |
| Mitochondrial proteins | Rats | Liver | SARDH↑, CPS↑, DLD↑, PDH↓, α-KGDH↓, MCAD↓ | 34 |

SARDH: Sarcosine dehydrogenase, CPS: carbamyl phosphate synthase, DLD: Dihydrolipoamide dehydrogenase, PDH: Pyruvate dehydrogenase, α-KGDH: α Ketoglutarate dehydrogenase, MCAD: acyl CoA dehydrogenase, SDH: Succinate Dehydrogenase.

**References in supplementary tables**

1. Chen Z, Hu C, Pan X, Zhao L, Xiong R, Geng Y, Hu L. Effect of processed Buguzhi and Roudoukou on energy metabolism in rats with spleen-kidney yang deficiency diarrhea (Articles in Chinese). Chinese Traditional Patent Medicine (2015) 37:1298-1301.
2. Huang Y, Fan Y, Jia T, Geng T, Shi J. Effect of Morinda officinalis Root and Its Salt-Processing Product on Energy Metabolism in Rats with Yang Deficiency (Articles in Chinese). Journal of Chinese Medicinal Materials (2016) 39:1028-1031.
3. Qiu L, Zhao Q, Dai Z, Deng N, Liu X, Hu F. Study on the Compatibility Mechanism of "Shaohuoshengqi " in Shenqi Pills Based on Mitochondrial Energy Metabolism (Articles in Chinese). Lishizhen Medicine and Materia Medica Research (2019) 30:1850-1853.
4. Liu X, Zhou H, Rong J, Wang X, Guo W, Xue J, Yuan C, Shi W, Huang M, Qu H. Effect and mechanisms of warming Kidney on Myocardial Energy Metabolism of Heart Failure Rats (Articles in Chinese). Pharmacology and Clinics of Chinese Materia Medica (2013) 29: 180-182.
5. Lu D, Wo X, Shi M, Li Y, Tang L. Relativity of liver mitochondria proteome and energy metabolism in shenyang deficiency animals induced by hormone (Articles in Chinese). Chinese Journal of Biochemistry and Molecular Biology (2005) 21: 807-353.
6. Zheng H, Jiang J, Jia W, He m, Jiang M, Ruan X, Su M, Wu J. Research on Metabonomics in Chronic Heart Failure with Kidney-Yang Deficiency (Articles in Chinese). Chinese Journal of traditional Chinese Medicine and Pharmacy (2010) 25:198-201.
7. Chen R, Wang J, Zhan R, Zhang L, Wang X. Integrated Systems Pharmacology, Urinary Metabonomics, and Quantitative Real-Time PCR Analysis to Uncover Targets and Metabolic Pathways of the You-Gui Pill in Treating Kidney-Yang Deficiency Syndrome. Int J Mol Sci (2019) 20:3655.
